# Exploring Effects of Chitosan Oligosaccharides on Mice Gut Microbiota in *in vitro* Fermentation and Animal Model

**DOI:** 10.3389/fmicb.2018.02388

**Published:** 2018-10-09

**Authors:** Chen Zhang, Siming Jiao, Zhuo A. Wang, Yuguang Du

**Affiliations:** State Key Laboratory of Biochemical Engineering, Institute of Process Engineering, Chinese Academy of Sciences, Beijing, China

**Keywords:** chitosan oligosaccharides, mice gut microbiota, *in vitro* fermentation, SCFAs, bacterial community

## Abstract

Chitosan oligosaccharides (COS) have shown positive effects on host gut health and influence on intestinal microbial community. However, the bioactivity and mechanism of COS on gut microbiota is still poorly understood. Here, we presented systematic studies of COS on mice fecal/gut microbiota. During *in vitro* fermentation of COS by mice gut microbiota, total bacterial population significantly decreased after 8-h COS treatment but was returned to the normal level after extended incubation. Consumption of COS and production of SCFAs suggested that COS were utilized by the microbe, although the consumption of chitosan pentasaccharides was obviously slower than others. COS treatments on mice fecal samples caused the decrease of potential pathogenic genera *Escherichia/Shigella* and the increase of genus *Parabacteroides*. *In vivo* animal study indicated that COS reduced population of probiotic genera *Lactobacillus*, *Bifidobacterium* and harmful genus *Desulfovibrio*, and increased abundance of genus *Akkermansia*. Phylum *Proteobacteria* was significantly inhibited by COS both in the animal model and *in vitro* fermentation. Our findings suggested that COS could reform the community structure of gut microbiota. The relationship among COS, gut microbiota and host health deserve further study.

## Introduction

Chitin the second most abundant biopolymer in the world. Chitosan oligosaccharides (COS), a mixture of oligomers of beta-1, 4-linked D-glucosamine, are prepared from chitosan, a N-deacetylated derivative is of chitin (Thadathil and Velappan, 2014). The water-soluble and low toxicity of COS assists their versatile biological activities (Muanprasat and Chatsudthipong, 2017). COS have been reported to possess various properties like anti-microbial (Choi et al., 2001), anti-inflammatory (Yousef et al., 2012), anti-diabetic activities (Lee et al., 2003), etc. Therefore, COS have good applicable values in food (Du et al., 2009) and pharmaceutical industries (Berger et al., 2004).

Gut microbiota can be considered as an extra organ with remarkable dynamics that influence the host gut health. Thousands of genes that encode carbohydrate-active enzymes (CAZymes) have been identified in the human gut microbiome (El Kaoutari et al., 2013). However, the human genome encodes, at most, only 17 enzymes for the digestion of food glycans, specifically starch, sucrose, and lactose and no polysaccharide lyases (Cantarel et al., 2012). COS as potential non-digestible oligosaccharides for the host, could be metabolized by gut microbiota. Dietary supplementation of COS has been found to improve gut barrier function, increase the population of *Bifidobacterium* spp. and *Lactobacillus* spp., and leave *Escherichia coli* counts unaffected in the cecum of weanling pigs (Yang et al., 2012). On the other hand, various studies reported that supplementation of COS significantly increased the abundance of *Escherichia* spp. in rats (Shang et al., 2017), but decreased the relative abundance of *Lactobacillus* in weanling pigs (Yu et al., 2017). Moreover, supplementation of COS and resistant starch mixtures alleviated metabolic disorders through synergistic actions, including positive manipulations on gut microbiota, lipid metabolism, and thickness of colonic mucosa layer in the rat (Shang et al., 2017).

Potential functions of oligosaccharides on gut microbiota could be tested using *in vitro* batch fermentation models that could exclude interferences from the host (Rastall, 2010; Payne et al., 2012). Limited number of studies had investigated the effect of COS on the gut microbiota *in vitro* and provided controversial conclusions. Lee et al. (2002) showed that COS stimulated the growth of *Bifidobacterium bifudium* and *Lactobacillus* spp. in pure cultures. On the contrary, Vernazza et al. (2005) did not observe an increase in *Bifidobacteria* counts in human fecal samples under COS treatments. This inconsistency through different researches might be due to different sources of COS and experimental settings (Lee et al., 2002; Mateos-Aparicio et al., 2016; Zou et al., 2016).

Based on these studies, the relationship among COS, gut microbiota and host gut health had been preliminarily established. However, the mechanism and causality of this relationship was still poorly explored. As an important part of the mechanism, the direct impact of COS on the gut microbiota needed to be further studied.

To investigate the effect of COS on the gut microbiota, we have determined the structure of microbiota in mice feces incubated with COS in *in vitro* batch cultures and colon contents in mice feeding with COS. To achieve this goal, COS with determined degree of polymerization (DP) and deacetylation (DD) was prepared. Batch cultures of mice feces were carried out with different concentrations of COS during *in vitro* anaerobic incubation. Consumption of COS as well as production of short chain fatty acids (SCFAs) and biogas were determined. Furthermore, the influence of COS upon both fecal microbiota *in vitro* and gut microbiota in animal was assessed. These studies are an important step toward a better understanding of the COS–microbe–host relationship.

## Materials and Methods

### Preparation and Identification of Chitosan Oligosaccharide

Chitosan oligosaccharide with the deacetylation degree over 95% and average molecular weight below 1 kDa were prepared as previously described (Zhang et al., 1999). The content of COS was determined by UPLC-ESI-Q-TOF-MS (Waters Corp., Milford, MA, United States) equipped with an Acquity HSS T3 column (100 mm × 2.1 mm, 1.8 μm) (**Supplementary Figure S1**), showing a range of DP from 2 to 6. COS with different DP from 2 to 6 were shown as COS-2, COS-3, COS-4, COS-5, and COS-6, respectively. Aqueous solutions (5 μL) of COS (10 mg mL^-1^) were injected into HPLC for analysis. Mobile phase was composed of solution A (10 mM ammonium formate in water) and solution B (acetonitrile) with a gradient elution as follows: from 0 to 2 min, 15% (A) 85% (B); from 2 to 32 min, 15–50% (A) 85–50% (B); and from 32 to 33 min, 50–80% (A) 50–20% (B) at the flow rate of 0.3 mL min^-1^. Mass spectrometry was applied for the qualitative analysis under negative ion modes. The MS parameters were set up as follows: capillary voltage, 3 kV; cone voltage, 30 V; source temperature, 120°C; desolvation temperature, 550°C; gas flows of cone and desolvation, 50 and 1000 L h^-1^. Mass spectrometry data were processed using Masslynx 4.1 software. COS samples were also analyzed using Acchrom S6000 HPLC system (Acchrom, China) equipment with an Acchrom XAmide column (4.6 mm × 250 mm × 5 μm, Acchrom, China) coupled with the evaporation light-scattering detector. Mobile phase was composed of acetonitrile (A) and 100 mM ammonium formate (B) with a gradient elution as follows: from 0 to 25 min, 90% (A) 10% (B); then from 26 to 70 min, 70% (A) 30% (B). The flow rate was 0.5 mL min^-1^ and the column temperature was 30°C. The relative abundance of COS oligomers was evaluated by determining the peak area of each oligosaccharide component using COS oligomer standards as external standard (Dalian Glycobio Co., Ltd.). Each sample was analyzed for three times.

### Anaerobic Incubation

Fresh feces (0.25 g) of male C57BL/6J mice were uniformly dispersed by vortex to get seed liquid in 50 mL a rich gut microbiota medium (GMM), which was prepared as described earlier (Goodman et al., 2011; Zhang et al., 2014). Anaerobic incubations were prepared by adding 3 mL of seed liquid into 27 mL of fresh GMM in 100-mL glass bottles. The stock solution of COS (100 g L^-1^) was sterilized by filtration, then added into samples to final concentrations of 0.1, 1, and 3 g L^-1^, respectively. Same volume of GMM was added to the control treatment. Bottles were sealed with butyl stoppers, then flushed with N_2_ for 3 min and incubated at 37°C. After 72 h incubation, 3 mL cultured samples of the primary culture (G1) were transferred into the 27 mL GMM to get the second subculture (G2). The 3rd subculture (G3) was conducted after 168 h incubation of the G2. All tests were carried out in triplicate for each treatment.

### Gas and Short Chain Fatty Acids (SCFAs) Analysis

Gaseous samples (100 μL) were collected from the headspace of the bottle and injected into GC with a pressure-lock precision analytical syringe (Baton Rouge, LA, United States) and concentrations of H_2_, CH_4_, and CO_2_ were analyzed using a gas chromatograph GC-7890A (Agilent Technologies, United States), equipped with thermal conductivity detector (TCD), flame ionization detector (FID) and Electrical Conductivity Detector (ECD) (Zhang et al., 2014). Fermentation samples were centrifuged at 14,000 ×*g* for 10 min and the supernatants were filtered through 0.22 μm (pore size) filters (Sangon, Shanghai, China) and stored at -20°C. The sediments were stored at -80°C for DNA extraction. SCFAs including formate, acetate, butyrate and L-lactate were analyzed using an Acchrom S6000 high performance liquid chromatography (HPLC) system (Acchrom, China) equipment with a C18 column (4.6 mm × 250 mm × 5 μm, Acchrom, China) at 210 nm of UV absorbance. Five microliter sample was injected into the HPLC system for each run. Mobile phase was composed of 1% acetonitrile and 99% NaH_2_PO_4_ with isocratic elution. The pH of mobile phase was adjusted to 2.0 with phosphoric acid.

### Nucleic Acid Extraction and Quantitative Analysis of Bacteria

Cultured samples were taken from incubations at indicated time points. Microbial DNA was extracted following the protocol of FastDNA SPIN Kit with bead-beating using FastPrep-24 (MP Biomedicals, CA, United States). The copy number of bacterial 16S rRNA gene was determined using quantitative PCR in a StepOne real-time PCR system (Applied Biosystems) with the primer pair Ba519f (CAGCMGCCGCGGTAANWC) and Ba907r (CCGTCAATTCMTTTRAGTTT) (Lueders et al., 2004). Standards with a 10-fold dilution series from 8.07 × 10^8^ to 8.07 × 10^3^ copies μL^-1^, were prepared from the purified plasmid DNA carrying bacterial 16S rRNA gene of *Allobaculum stercoricanis* (DSM 13633T).

### Animal Feeding Trial

Male C57BL/6J (6 weeks old) mice were purchased from Model Animal Research Center of Nanjing University (Nanjing, China). After acclimation for 1 week, 10 mice were randomly divided into two groups (*n* = 5): chow diet (CD) group and CD + COS (1 g L^-1^ in drinking water, about 200 mg kg⋅d^-1^) group. The standard CD diet (Aoke Xieli Co., Ltd., China) contained 20% protein, 70% carbohydrate, and 10% fat (**Supplementary Table S1**). After feeding for 5 months at 22°C with a 12–12 h dark–light cycle, all mice were euthanized and colon contents were sampled. Samples were immediately frozen in liquid nitrogen and stored at -80°C until DNA extraction. The animal experiments were approved by the Animal Ethical Experimentation Committee of Chinese Academy of Sciences (permission number: SYXK2015-0002).

### Sequencing and Bacterial Community Analyses

The V3–V4 region of bacterial 16S rRNA genes was subjected to PCR amplification. Purified amplicons were sequenced on the Illumina MiSeq platform by Allwegene Technology Inc. (Beijing, China). Raw sequences were analyzed by QIIME software package as described previously (Caporaso et al., 2010). Unique sequences were clustered into operational taxonomic units (OTUs) defined by 97% similarity. Alpha and beta diversity were examined as well as redundancy analysis (RDA) and principal coordinate analysis (PCoA). Linear discriminant analysis effect size (LEfSe) was used to estimate taxonomic abundance and characterize differences between groups (Segata et al., 2011). All 16S rRNA pyrosequencing datasets had been deposited in GenBank Sequence Read Archive (SRA) database (Accession number: SRP114727 and SRP142222).

### Statistical Analysis

All statistical analyses were carried out using GraphPad Prism (version 6, GraphPad Software Inc., San Diego, CA, United States). Data were presented as means ± SD. Data of the H_2_ and SCFAs concentrations and bacterial population abundance were subjected to the parametric ANOVA analysis, along with the Tukey–Kramer test, to determine the significant differences between the treatments. LEfSe analysis of the treatment groups was performed on the basis of the results of the Kruskal–Wallis and Wilcoxon tests and the threshold on the logarithmic linear discriminant analysis (LDA) score was 2.0. The *P*-value < 0.05 was regarded as statistical significance.

## Results

### *In vitro* Fermentation of COS in Mice Fecal Samples

**FIGURE 1:**
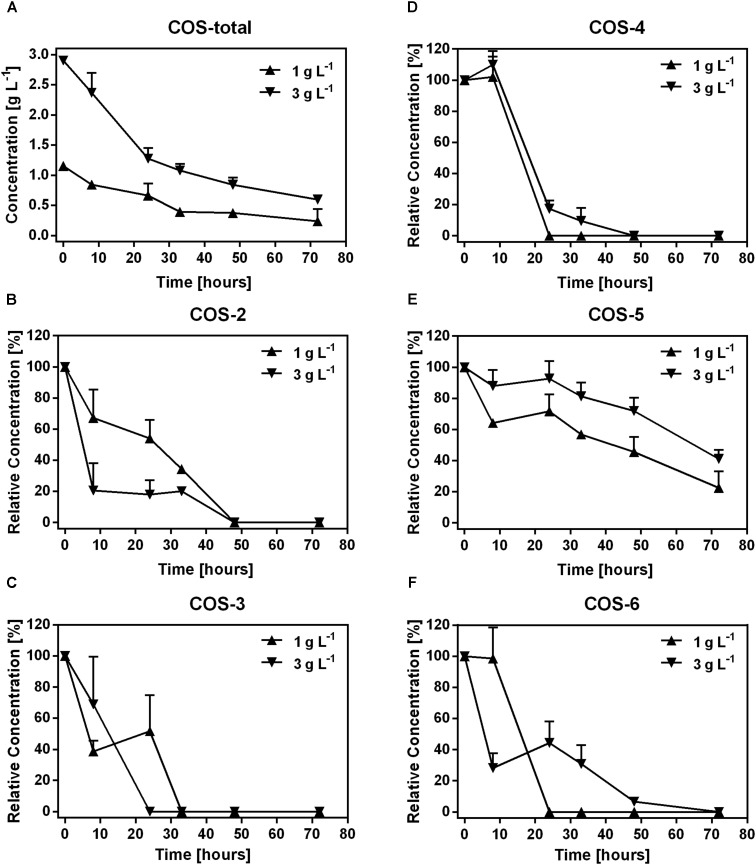
Dynamics of COS polymers during COS treatment on mice feces. The concentration of COS-total **(A)**, and the level of COS-2 **(B)**, COS-3 **(C)**, COS-4 **(D)**, COS-5 **(E)**, and COS-6 **(F)** in mice feces treated with 0.1, 1, and 3 g L^-1^ COS, respectively. COS with different degree of polymerization (DP) from 2 to 6 were shown as COS-2, COS-3, COS-4, COS-5, and COS-6, respectively. Data were collected at 0, 8, 24, 32, 48, and 72 h of the treatment. Data are represented as the means ± SD (*n* = 3).

In the first 33 h, 0.76 g L^-1^ and 1.82 g L^-1^ COS were degraded in samples treated with 1 and 3 g L^-1^ COS, respectively. Then, the degradation was slow down with only 0.15 g L^-1^ and 0.48 g L^-1^ COS were utilized in the following time (**Figure 1A**). The change of COS was not recorded in samples treated with 0.1 g L^-1^ COS since the concentration was below the detection limit (about 0.1 g L^-1^) of the HPLC instrument used in the experiment. To determine the degradation of different COS components by mice fecal microbiota, changes in the relative abundance of each COS oligomer were characterized through fermentation. Contents of major components of the original COS sample were COS-2 (DP2) 3.4%, COS-3 (DP3) 17.8%, COS-4 (DP4) 32.1%, COS-5 (DP5) 25.9%, and COS-6 (DP6) 12%, respectively, accounted for 91.2% of total COS (**Supplementary Figures S1**, **S2**). During fermentation, concentrations of COS-2, 3, 4, and 6 all dropped quickly to undetectable level (<0.1 g L^-1^ COS) after 48 h (**Figures 1B–D,F**). Interestingly, degradation of COS-5 was obviously slower than other components (**Figure 1E**). Till the end of the incubation, almost 46 and 72% of COS-5 still remained in the treatments with 1 and 3 g L^-1^ COS, respectively.

### H_2_, Lactate and SCFAs Dynamics

**FIGURE 2:**
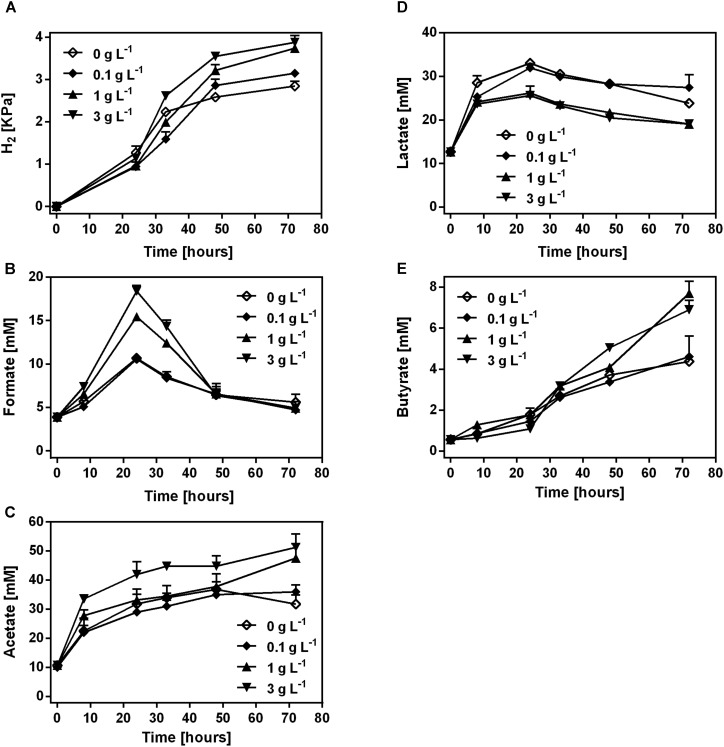
Production of H_2_ and SCFAs by the mice fecal microbe during COS treatment. The concentration of H_2_ **(A)**, formate **(B)**, acetate **(C)**, lactate **(D),** and butyrate **(E)** was analyzed in mice feces treated with 0, 0.1, 1, or 3 g L^-1^ COS, respectively. Data were collected at 0, 8, 24, 32, 48, and 72 h of the treatment. Data are represented as the means ± SD (*n* = 3).

H_2_, lactate and SCFAs were produced during the fermentation of COS in mice fecal samples (**Figure 2**). H_2_, acetate and butyrate showed a continuous accumulation in the samples with COS treatment (**Figures 2A,C,E**). The concentration of H_2_, acetate and butyrate was significantly higher in treatments with 1 and 3 g L^-1^ COS than that in 0 and 0.1 g L^-1^ COS treatments at 72 h, respectively (**Supplementary Figures S3A,C,E**). At the end of incubation, the partial pressure of H_2_ was higher in COS treated samples (3.1–3.9 kPa) than that in the control (2.8 kPa). Acetate was the most abundant SCFAs and reached 36, 47.6, and 51.3 mM during the incubation in treatments with 0.1, 1, and 3 g L^-1^ COS, respectively. In the control, acetate increased to 36.8 mM at 48 h, and then decreased to 31.7 mM at the end of incubation (**Figure 2C**). Formate accumulated during the first 24 h, then decreased to about 6.5 mM after 48 h in all tests. The highest concentrations of formate were 15.4 and 18.4 mM in fecal samples with 1 and 3 g L^-1^ COS, respectively. However, the accumulation of formate only reached a relative low level in the control and 0.1 g L^-1^ COS treated groups (**Figure 2B** and **Supplementary Figure S3B**). In addition, the production of propionate could not be detected during the incubation.

In all tested groups, lactate accumulated during the first 24 h, then dropped till the end of the incubation (**Figure 2D**). The highest concentration of lactate was above 32 mM in the control and samples treated with 0.1 g L^-1^ COS, but only 26 mM in 1 and 3 g L^-1^ COS treated groups. Thus, the lactate production was significantly inhibited by COS in relative high concentrations (**Supplementary Figure S3D**). It suggested that lactate-producing microbe in the fecal sample was inhibited by COS.

### Qualification of Bacterial Community

The total bacterial 16S rRNA gene copy numbers were estimated by quantitative PCR (**Table 1**). After 8 h incubation, the copy numbers of bacterial 16S rRNA gene significantly decreased from 3.5 × 10^11^ copies mL^-1^ to 1.1 × 10^9^ copies mL^-1^ and 2.9 × 10^8^ copies mL^-1^ in the 1 g L^-1^ COS and 3 g L^-1^ COS treated samples, respectively. However, copy numbers of bacterial 16S rRNA gene under these treatments returned to the same level as the control group after 72 h incubation. Besides, the bacterial copy numbers were barely affected by the treatment with 0.1 g L^-1^ COS. Thus, the inhibitory effect of COS on the fecal bacteria was dependent on the concentration of COS as expected. However, the population of the bacterial community quickly recovered with the extended incubation time likely due to the degradation of COS as shown above.

**Table 1:** Abundance of bacterial population through COS treatment.

COS Concentration [g L^-1^]	Log_10_ [Bacteria 16S rRNA gene (copies mL^-1^)]		
	Inocula	8 h	72 h
0	11.32 ± 0.07	11.54 ± 0.08^a^	12.30 ± 0.08^ab^
0.1		11.10 ± 0.1^b^	12.35 ± 0.08^a^
1		9.02 ± 0.1^c^	12.27 ± 0.07^ab^
3		8.44 ± 0.1^d^	12.12 ± 0.06^b^

*Abundance of bacteria was estimated by quantitative PCR analyses based on bacteria 16S rRNA gene copy numbers in the liquid medium containing mice feces treated with 0, 0.1, 1, or 3 g L**^-^**^1^ COS, respectively. Data were collected at 0, 8, and 72 h of the treatment. Data are represented as the means ± SD (n = 3). Superscripts with different letters indicate significant differences (P < 0.05) between treatments at the same time point.*

### Change of Bacterial Community During *in vitro* Fermentation of Mice Feces With COS

**FIGURE 3:**
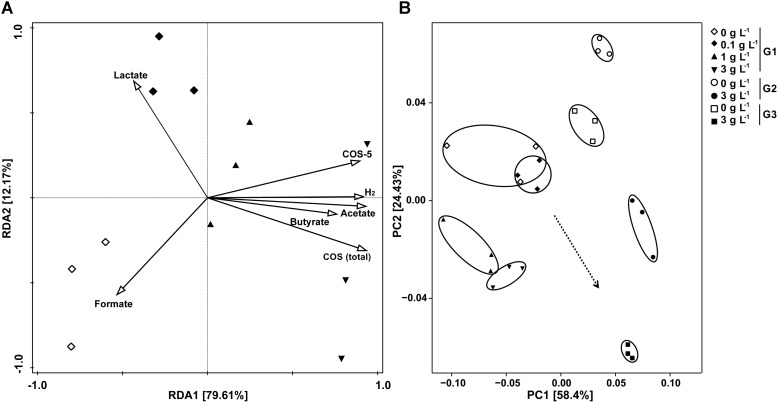
Redundancy analysis (RDA) and UniFrac-based PCoA analysis on bacterial diversity influenced by COS treatment. RDA for samples from primary culture (G1) with COS (total), COS-5, H_2_, SCFA and acetate as environmental variables **(A)**. The length of each arrow indicated the contribution of the corresponding parameter to the structural variation. UniFrac-based PCoA using the weighted version for samples from the primary culture and subculture **(B)**. Mice stool incubated with 0, 0.1, 1, or 3 g L^-1^ COS at 72 h of the primary culture (G1), and 0 or 3 g L^-1^ COS at the end of the 2nd subculture (G2), and 0 or 3 g L^-1^ COS at the end of the 3rd subculture (G3).

To determine the composition of the bacterial community, bacterial 16S rRNA gene pyrosequencing was performed. Alfa-diversity of bacterial community of samples showed that, as the sequencing depth increased, the number of observed species also increased (**Supplementary Figure S4A**). There was no significant difference in richness (as indicated by rarefaction of observed species) and diversity between the control and samples treated with 0.1 g L^-1^ COS, while addition of >1 g L^-1^ COS significantly reduced both the richness and diversity of the bacterial community (**Supplementary Figure S4B**). RDA was conducted to examine the relationships between environmental properties and bacterial population distribution of the samples (**Figure 3A**). Content change of COS, COS-5, H_2_, acetate and butyrate explained 79.6% of the total variance of the bacterial population, while lactate and formate were related to 12.2% of the total variance. The concentration of COS was positively associated with the H_2_ partial pressure and the concentration of acetate and butyrate. However, there was a negative correlation between COS and lactate concentration. UniFrac-based principal coordinates analysis (PCoA) revealed a distinct clustering of bacteria composition for each experimental group (**Figure 3B**). Those results indicated that the addition of more than 1 g L^-1^ COS markedly affected bacterial population distribution.

**FIGURE 4:**
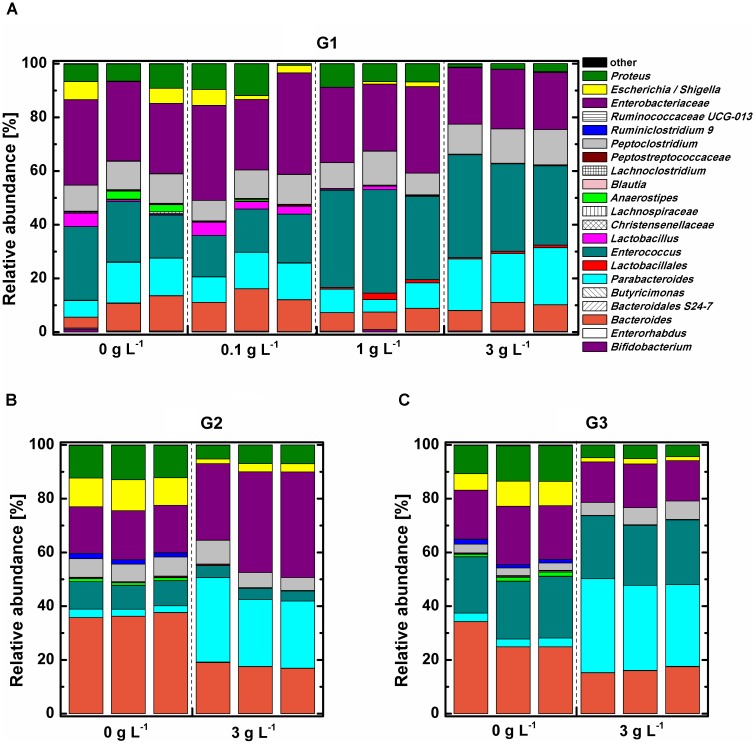
Relative abundance of bacterial community in mice fecal samples at genus level. Mice fecal samples treated with 0, 0.1, 1, 3 g L^-1^ COS at 72 h of the primary culture (G1) **(A)**, 0 or 3 g L^-1^ COS at the end of the 2nd subculture (G2) **(B)**, and 0 or 3 g L^-1^ COS at the end of the 3rd subculture (G3) **(C)**.

Phylogenetic analysis indicated that all tested samples comprised mainly phylum *Proteobacteria*, *Firmicutes*, *Bacteroidetes*, and *Actinobacteria* (**Supplementary Figure S5**). The abundance of the phylum *Bacteroidetes*, which was dominant with genus *Parabacteroides*, was the significantly increased (*P* < 0.05), while phylum *Proteobacteria* was remarkably decreased (*P* < 0.05) under 3 g L^-1^ COS treatment at the end of the 3rd subculture. The influence of COS on the relative abundance of bacterial community at the genus level was analyzed (**Figure 4**). The bacterial community structure in the 0.1 g L^-1^ COS-treated group remained similar to the control after 72 h of incubation. *Enterobacteriaceae*, accounting for about 30% of total bacterial abundance, predominated in both groups. *Enterococcus* accounted for 16–22% of total bacterial abundance. *Enterococcus* was further enriched in samples treated with more than 1 g L^-1^ COS and accounted for 33–35% of total bacterial population. In addition, *Lactobacillus*, *Escherichia/Shigella* decreased in abundance from 4.17% to 0.02–0.69% after COS treatments (>1 g L^-1^). The decreased abundance of *Lactobacillus* indicated the inhibitory effect of COS (>1 g L^-1^) on the growth of genus *Lactobacillus*, consistent with the experimental result of COS treatment on the growth of three different *Lactobacillus* strains in pure cultures (**Supplementary Figure S8**).

The trend of changed proportions of certain bacterial groups in the mice fecal sample under COS treatment was further intensified by subcultures (**Figures 4B,C**). The relative abundance of *Proteus*, *Escherichia/Shigella*, and *Bacteroides* decreased 2–5-fold in 3 g L^-1^ COS treated group. Furthermore, COS significantly stimulated the growth of *Parabacteroides* through transfer of subcultures. The relative abundance of *Parabacteroides* was 10 times higher in 3 g L^-1^ COS treated group than that in the control.

Linear discriminant analysis (LDA) effect size (LEfSe) was performed to identified bacteria strains which exhibited obvious changes in relative abundance under COS treatment (**Supplementary Figures S6, S7**). Six different taxa were identified in samples treated with 1 g L^-1^ COS and 10 taxa in samples with 3 g L^-1^ COS (**Supplementary Figures S6A,B**). COS treatments significantly increased proportions of *Enterococcus* and *Lactobacillales* (**Supplementary Figures S6E,F**). On the contrary, the abundance of *Enterorhabdus* and *Ruminiclostridium*_9 significantly dropped under COS treatments. It is important to note that the relative abundance of *Lactobacillus* decreased significantly from 2 to 0.02% under 3 g L^-1^ COS treatment.

### *In vivo* Feeding Trial

**FIGURE 5:**
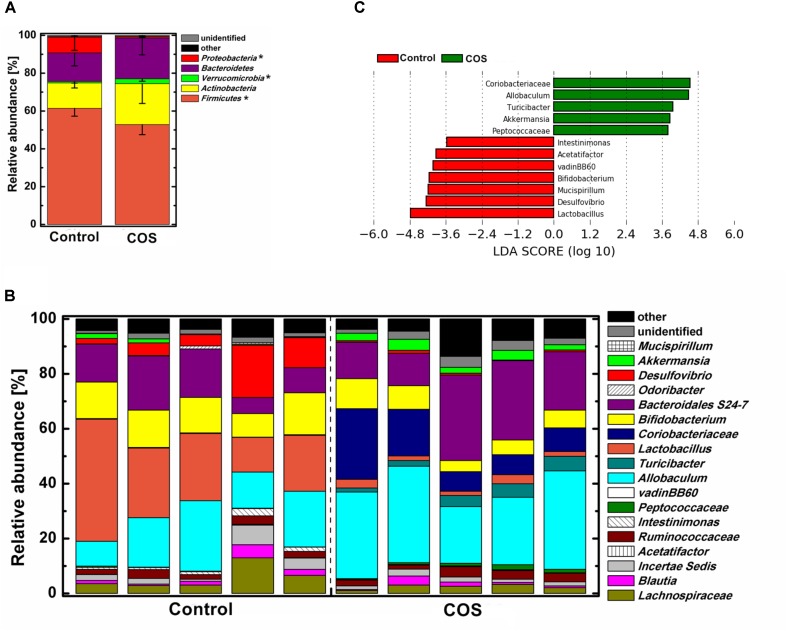
Effect of COS treatment on the structure of gut bacterial community in mice. Relative abundance of bacterial community at phylum level **(A)** genus level **(B)**. Significant differences of the bacteria taxa in mice with or without COS treatments based on Linear discriminant analysis effect size (LEfSe) **(C)**. Significant differences in LDA scores (*P* < 0.05) were produced among classes (Kruskal–Wallis test) and between subclasses (Wilcoxon’s test). The threshold logarithmic LDA score was 3.6. Significant differences were noted as follows: ^∗^*P* < 0.05.

The gut microbiota was dominated by four major phyla, *Firmicutes*, *Actinobacteria*, *Bacteroidetes*, and *Proteobacteria* in the control mice (**Figure 5A**). With the supplementation of COS, the abundance of the phylum *Verrucomicrobia* was significantly increased (*P* < 0.05), while *Firmicutes* and *Proteobacteria* was remarkably decreased (*P* < 0.05). Moreover, the abundance of the phylum *Bacteroidetes* in the COS group showed a trend of increase, however, it was not significantly different from that in the control. At genus level (**Figures 5B,C**), there were several bacteria increased in the COS group (*P* < 0.05), including *Coriobacteriaceae*, *Allobaculum*, *Turicibacter*, *Akkermansia*, and *Peptococcaceae*. On the contrary, the abundances of *Lactobacillus*, *Desulfovibrio*, *Bifidobacterium*, *Mucispirillum*, vadinBB60, *Acetatifactor* and *Intestinimonas* were dramatically suppressed with the administration of COS (*P* < 0.05 or *P* < 0.01). In addition, the relative abundance of *Mucispirillum*, vadinBB60 and *Acetatifactor* were below 0.5% in the COS group.

## Discussion

Gut microbiota is associated with not only food digestion and metabolism, but also gut heath and several diseases (Clemente et al., 2012). COS have shown versatile health-related biological functions such as immune-stimulation, anti-microbial activity (Liaqat and Eltem, 2018). Those biological functions might be related to interaction with gut microbiota. An important result of our present studies is a comprehensive and comparative analysis of the microbial community from mice fecal samples treated with different concentrations of COS both *in vitro* and in animal model. Addition of 1 g L^-1^ or higher concentration of COS significantly inhibited the growth of fecal bacteria *in vitro*. However, following the elongation of the incubation time, the total number of bacteria was quickly returned to the same level of bacteria in the untreated control (**Table 1**), likely due to the consumption of COS by the microbe (**Figures 1**, **2**). Interestingly, the utilization rate was different among COS oligomers, in which COS-5 was obviously not favored by the fecal microbe. At the phylum level, the abundance of *Bacteroidetes* was the significantly increased, while *Proteobacteria* was remarkably decreased under 3 g L^-1^ COS treatment at the end of the 3rd subculture. Moreover, amounts of genera *Bacteroides*, *Proteus*, and *Escherichia/Shigella*, were significantly decreased and *Parabacteroides* spp. was increased under COS treatments *in vitro*. The trend was reinforced by increased concentration of COS and times of subculture. Changes on the production of several kinds of SCFA from *in vitro* fermentation were also identified. The relationship among COS, microbe and metabolites was also preliminarily established. In the mouse model, the abundance of phylum *Firmicutes* and *Proteobacteria* was remarkably decreased in the colon contents of mice fed with COS. At the genus level, supplementation of COS obviously increased the abundance of beneficial bacteria *Akkermansia*, and greatly decreased the relative abundance of harmful bacteria *Desulfovibrio* (phylum *Proteobacteria*).

Chitosan oligosaccharides have been shown to exhibit inhibitory effects against both Gram-positive and Gram-negative bacteria with the minimum inhibitory concentration (MIC) of 0.8–1.2 g L^-1^ (Kittur et al., 2005; Wang et al., 2007). Our studies also found that copy numbers of bacterial 16S rRNA genes in samples treated with 0.1, 1, and 3 g L^-1^ COS were decreased 2.7, 300, 1200 times compared to the control, respectively. However, there was no significant difference in 16S rRNA genes copy numbers between the COS treated groups and the control after incubation for 72 h. Same trend was observed when *Lactobacillus* spp. strains were treated with COS in pure cultures (**Supplementary Figure S8**). Thus, the inhibitory effects of COS on bacteria declined in accordance with the extension of the treatment time. Consistently, the concentration of COS in the sample dropped down quickly (**Figure 1**), indicating the consumption of COS by the microbe. In addition, cellulose as a limited fermentability control is helpful for confirming COS as microbiota-accessible carbohydrates.

Degree of polymerization of COS is tightly associated with its bio-activities (Kittur et al., 2005; Mateos-Aparicio et al., 2016). In the present study, COS oligomers showed different degrading dynamics by the fecal microbe: slower degradation of COS with higher DP (**Figure 1**). The transient accumulation of COS with lower DP might be due to the degradation of larger oligomers. Interestingly, degradation of COS-5 was obviously slower than others, suggesting the utilization of COS-5 was relatively difficult or unfavorable for the microbe (**Figure 1E**). In fact both direct cellular uptake and extracellular enzymatic degradation might contribute to COS consumption by gut microbe. Studies showed that gut bacteria was able to directly uptake a variety of oligosaccharides through specific transporters (Abou Hachem et al., 2013). A recent study showed that cell uptake of COS dimer (COS-2) was faster than trimer (COS-3) by *S*. *coelicolor* (Viens et al., 2015), suggesting lower DP was more favorable for the bacteria. Furthermore, chitosanase have been found in a variety of microorganisms, and was usually expressed extracellularly (Jo et al., 2003; Liu et al., 2015). Interestingly, studies on GH8 and GH46 chitosanase families indicated that the substrates associated with them were both composed of six subsites (Liu et al., 2015). Moreover, the rate of COS degradation by some chitosanase obviously decreased with a decrease in the substrate size, with a 20-fold difference on the degradation rate between COS-6 and COS-5 (Jo et al., 2003). Thus, COS-5 might be unfavorable for both extracellular chitosanase degradation and cellular uptake of gut microbe. This observation brought an important proof for the structural and functional specificity of each COS oligomer. Thus, to better understand the structural-functional relationship of COS, purified COS oligomers should be used in the future studies.

Genera *Bacteroides* and *Proteus* and *Escherichia/Shigella* were significantly inhibited by COS treatment (**Supplementary Figure S6**). The inhibitory effect was enhanced with increased concentration of COS. Metagenomic studies had noted associations between certain species of *Bacteroides* and diabetes (Johnson et al., 2017). Qin et al. (2012) observed the genus *Bacteroides* was more abundant in type II diabetic cohorts and Karlsson et al. (2013) got the similar results. The *Proteus* spp. bacteria were mostly known as opportunistic human pathogens associated with complicated urinary tract and wound infections as well as nosocomial infections (Armbruster and Mobley, 2012). *Escherichia/Shigella* were leading pathogens, which could cause diarrhea worldwide. On the other hand, the relative abundance of *Parabacteroides* spp. was significantly increased under 3 g L^-1^ COS treatments (**Supplementary Figure S6**). Members of the phylum *Bacteroidetes*, including genus *Parabacteroides*, participated in provisioning the host with energy harvested through the fermentation of indigestible polysaccharides to produce SCFAs (Johnson et al., 2017), and were also related to butyrate production (Palakawong Na Ayudthaya et al., 2018). Moreover, colonic hydrogen concentration in rats was positively correlated with the abundance of genus *Parabacteroides* (Nishimura et al., 2018). Those results indicated that *Parabacteroides* spp. might contribute to the increased concentration of hydrogen and SCFAs through COS treatments (**Figures 1**–**3**). Butyrate was an important substrates in the degradation of non-digestible carbohydrates, rather than by protein fermentation under anaerobic conditions (Louis and Flint, 2017). During the anaerobic incubation, we did not detect the production of methane by GC analysis. In addition, there was no electron acceptors, such as nitrate or sulfate in the culture medium. Therefore, butyrate significantly accumulated in the *in vitro* incubation system. In the host *in vivo* environment, SCFAs reaching total concentrations of 50–200 mM in the human large intestine could be taken up efficiently by the gut mucosa and have important positive impacts upon host physiology (Koh et al., 2016; Louis and Flint, 2017). Especially, butyrate could be used preferentially as an energy source by the gut mucosa and most often considered to benefit health, including protection against colorectal cancer (Koh et al., 2016; Morrison and Preston, 2016). Thus, these results suggested that COS could positively manipulate the composition of microbial community and stimulate the production of SCFAs, which might bring benefits to gut health.

In animal study, COS as a potential non-digestable oligosaccharide for the host, could be metabolized by gut microbiota. Supplementation of COS greatly decreased the abundance of phylum *Proteobacteria* (**Supplementary Figure S5**), consistent with the result of *in vitro* study. The relative abundance of genus *Desulfovibrio* (phylum *Proteobacteria*), which may contribute to colorectal cancer development (Carbonero et al., 2012), greatly decreased with the supplementation of COS (**Figure 5**). Supplementation of COS also significantly inhibited probiotic genus *Lactobacillus* (**Figure 5**), consistent with the result of *in vitro* study. The probiotic genus *Bifidobacterium* was also significant inhibited in mice fed with COS. These results were consistent with previous observations (Mateos-Aparicio et al., 2016), and might indicate that COS should not be considered as traditional prebiotic. However, COS greatly elevated the abundance of beneficial genus *Akkermansia* in mice gut (**Figure 5**). Genus *Akkermansia* was involved in the enterocytes proliferation of the colonic wounds and reversing high-fat diet induced metabolic disorders, including fat-mass gain, adipose tissue inflammation and insulin resistance (Everard et al., 2013; Alam et al., 2016). It has to be noted that those beneficial effects of bacteria *Akkermansia* were in accordance with the biological activities of COS (Liaqat and Eltem, 2018). With the establishment of the exact role of intestinal microbial flora and the increasing species of probiotics, the scope and definition of prebiotics would be expanded and revised in the future (Bindels et al., 2015). Based on the beneficial effects of COS on host, it might be considered to be a kind of potential prebiotics. In addition, supplementation of COS increased the abundance of *Coriobacteriaceae*, which exhibited importance functions, such as the conversion of bile salts and steroids as well as the activation of dietary polyphenols (Clavel et al., 2006; Ridlon et al., 2006). Phylum *Proteobacteria* was significantly inhibited by COS both in the animal model and *in vitro* fermentation. However, at the genus level, structures of bacterial community were relative different between the animal model and *in vitro* fermentation. The difference between *in vivo* and *in vitro* environments led different effects of COS on gut microbiota.

Thus, COS might positively reform the composition of microbial community in both *in vitro* fermentation and animal studies, although affected bacteria taxa were difference between two experimental conditions. These results indicated the potential beneficial effects of COS on the host health through reforming the structure of intestinal bacteria.

## Conclusion

We reported the effects of COS on intestinal bacteria of mice *in vivo* and *in vitro*. During *in vitro* fecal cultivation, COS significantly inhibited the growth and diversity of total bacteria community at the beginning of incubation. The inhibitory effect of COS obviously declined with the utilization of COS by fecal microbe. The abundance of *Bacteroides* and *Escherichia*/*Shigella* decreased with the COS addition. However, COS could increase the abundance of *Parabacteroides*. In animal study, COS reduced the abundance of probiotic *Lactobacillus*, *Bifidobacterium* and harmful bacteria *Desulfovibrio*, and increased abundance of *Akkermansia*, which was in accordance with the beneficial activities of COS on host health. Phylum *Proteobacteria* was significantly inhibited by COS both in the animal model and *in vitro* fermentation. These results suggested that COS could result in substantial reform of the intestinal microbial community, which might contribute to the health status of the host.

## Author Contributions

CZ and YD participated in the design of the study. CZ conducted the experiments and analyzed the data. SJ analyzed the volatile fatty acids and chitosan oligosaccharides. CZ wrote the first draft of the manuscript. ZW revised the manuscript. All authors read and approved the final manuscript.

## Conflict of Interest Statement

The authors declare that the research was conducted in the absence of any commercial or financial relationships that could be construed as a potential conflict of interest.
