# Supplementary material for: Exploring Effects of Chitosan Oligosaccharides on Mice Gut Microbiota in in vitro Fermentation and Animal Model
Source: Front Microbiol. 2018 Oct 9;9:2388. doi: 10.3389/fmicb.2018.02388 (PMC6190755; doi:10.3389/fmicb.2018.02388)
Supplement: Supplementary file 1 [file Table_1.docx]

Table S1. Ingredients of control diet (CD) in the mice feeding experiment.

| Ingredient | Control Diet | |
| --- | --- | --- |
|  | Gram | Kcal |
| Casein, 80 Mesh | 200 | 800 |
| L-Cystine | 3 | 12 |
| Corn Starch | 315 | 1260 |
| Maltodextrin 10 | 35 | 140 |
| Sucrose | 350 | 1400 |
| Cellulose, BW200 | 50 | 0 |
| Soybean Oil | 25 | 225 |
| Lard | 20 | 180 |
| Mineral Mix S10026 | 10 | 0 |
| Dicalcium Phosphate | 13 | 0 |
| Calcium Carbonate | 5.5 | 0 |
| Potassium Citrate Monohydrate | 16.5 | 0 |
| Vitamin Mix V10001 | 10 | 40 |
| Choline Bitartrate | 2 | 0 |
| FD&C Red Dye #40 | 0.05 | 0 |
