# Supplementary material for: Exploring Effects of Chitosan Oligosaccharides on Mice Gut Microbiota in in vitro Fermentation and Animal Model
Source: Front Microbiol. 2018 Oct 9;9:2388. doi: 10.3389/fmicb.2018.02388 (PMC6190755; doi:10.3389/fmicb.2018.02388)
Supplement: Supplementary file 2 [file Image_1.pdf]

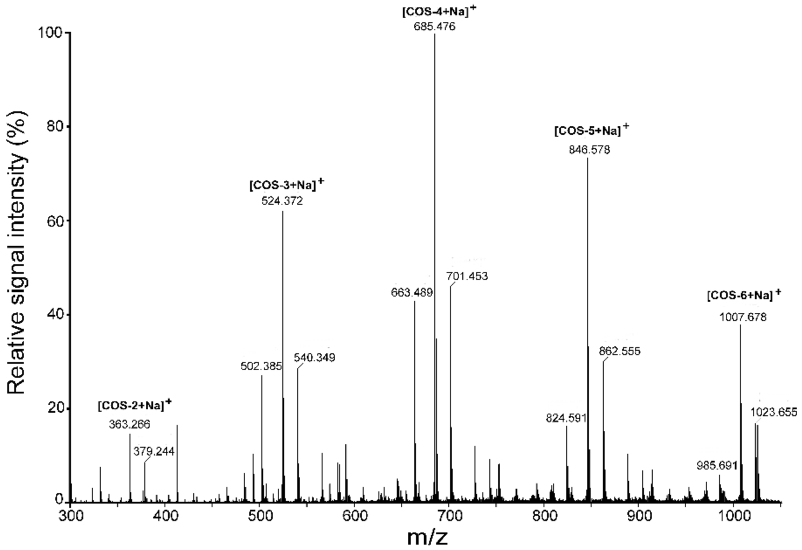

FIGURE S1: UPLC-Q-TOF mass spectrum of COS. Peaks indicate $[M + Na]^+$ of dimer COS-2 (363.366), trimer COS-3 (524.372), tetramer COS-4 (685.476), pentamer COS-5 (846.578), and hexamer COS-6 (1007.678).

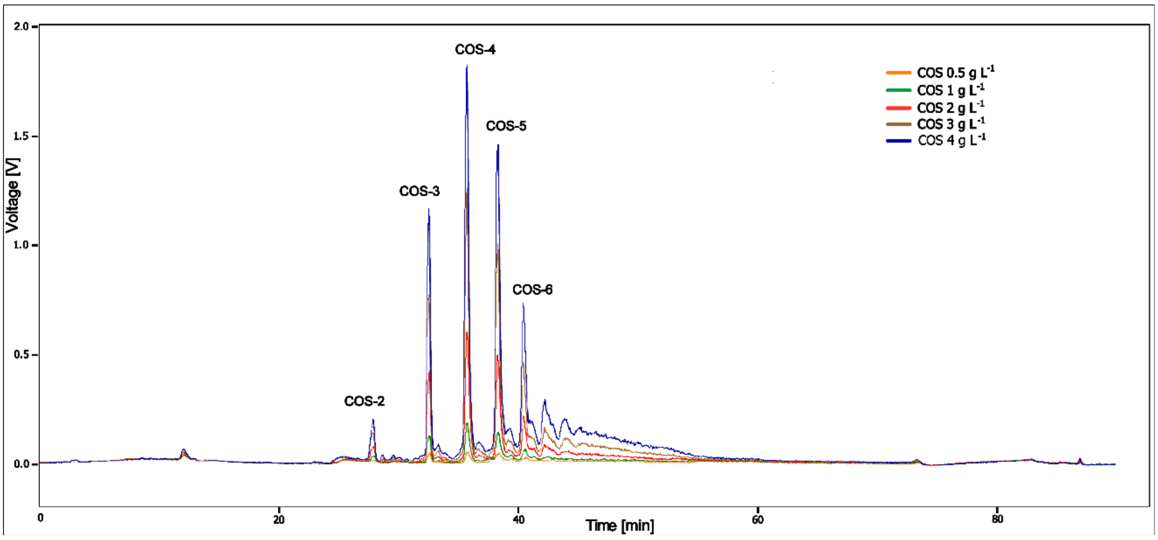

FIGURE S2: HPLC spectrum of COS. Peaks indicate of dimer COS-2, trimer COS-3, tetramer COS-4, pentamer COS-5, and hexamer COS-6 in different concentrations of COS samples.

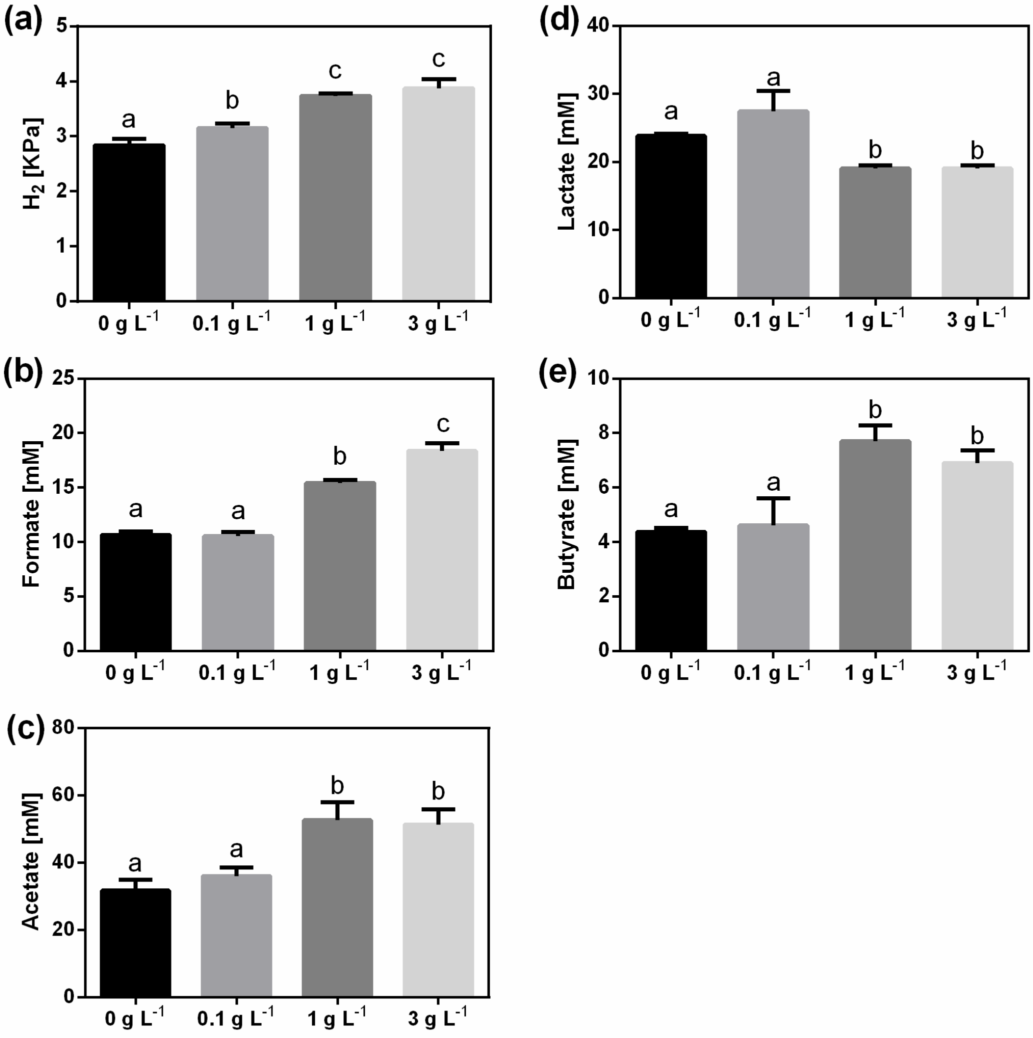

FIGURE S3: Statistical analysis of H_2 and SCFAs accumulation during COS treatment on mice feces. The concentration of H_2 at 72 hours (a), formate at 8 hours (b), acetate at 72 hours (c), lactate at 72 hours (d) and butyrate at 72 hours (e) was analyzed in mice feces treated with 0, 0.1, 1 or 3 g L^{-1} COS, respectively. Data are represented as the means ± SD (n=3). Different letters above the columns indicate a significant difference ($P < 0.05$).

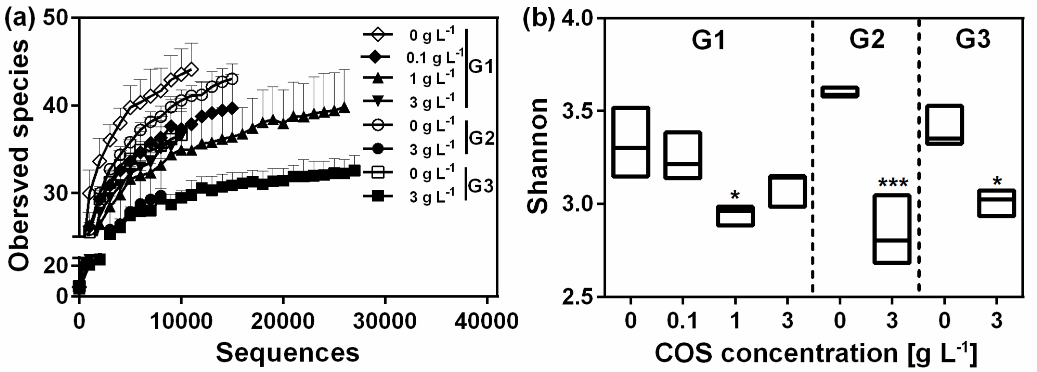

FIGURE S4: Diversity and richness of bacterial community. The x-axis shows the number of valid sequences per sample and the y-axis shows the observed species in rarefaction curves (a). Shannon values (b) of bacterial diversity was shown. Mice fecal samples treated with 0 g L^{-1} COS, 0.1 g L^{-1} COS, 1 g L^{-1} COS, 3 g L^{-1} COS at 72 hour of the primer culture, and 0 g L^{-1} COS, 3 g L^{-1} COS at the end of the 2nd subculture, and 0 g L^{-1} COS, 3 g L^{-1} COS at the end of the 3rd subculture. Differences were assessed by ANOVA and denoted as follows: * $P < 0.05$; ** $P < 0.01$; *** $P < 0.001$.

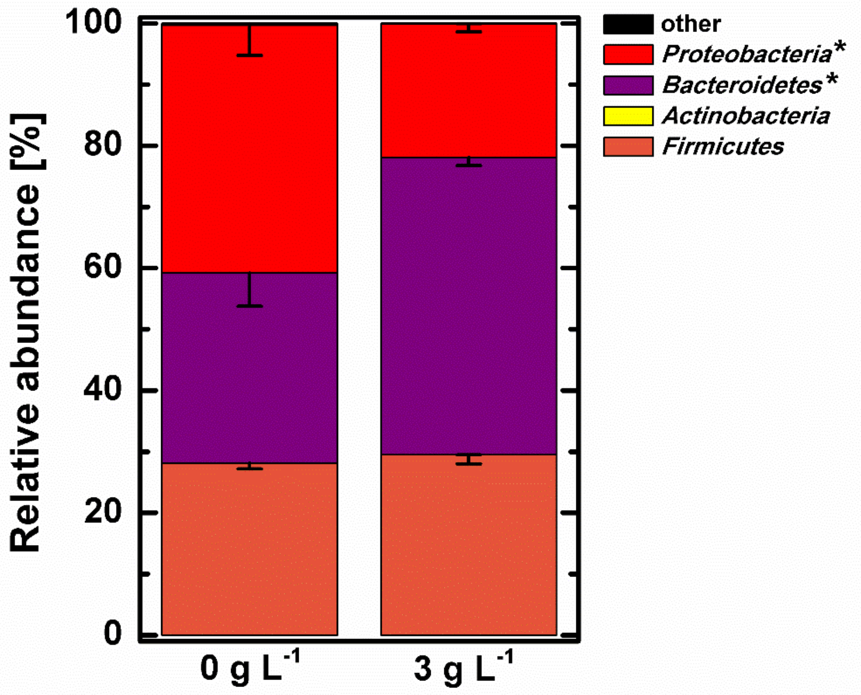

FIGURE S5: Relative abundance of bacterial community in mice fecal samples at phylum level at the end of the 3rd subculture. Significant differences were noted as follows: *$P < 0.05$.

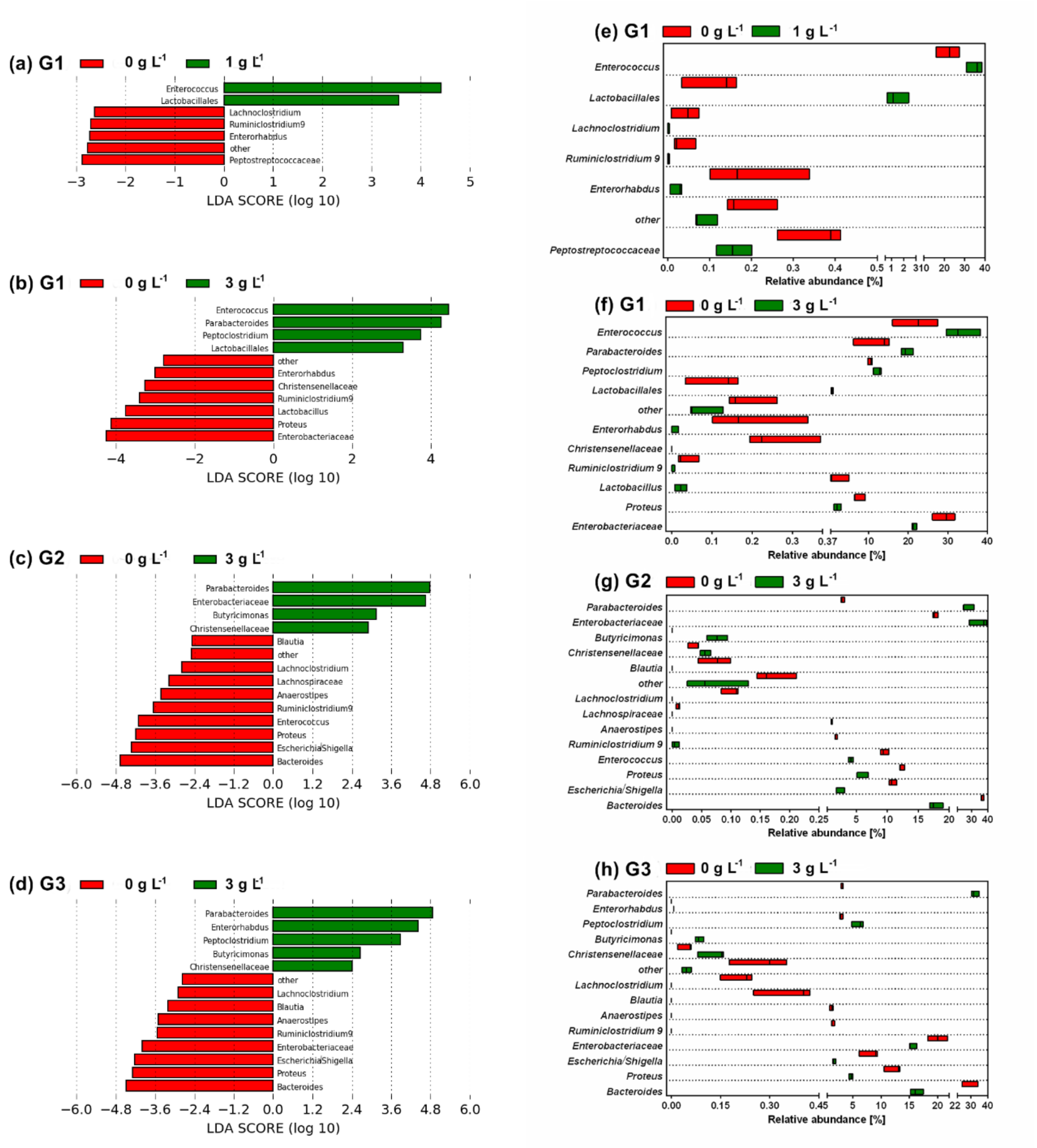

FIGURE S6: Significant differences in the bacteria taxa of mice fecal samples with or without COS treatment. A taxonomic comparison of the bacterial communities in samples with or without COS treatments based on Linear discriminant analysis effect size (LEfSe) (a-d) and the relative abundance of taxa (e-h) in the sample with COS (green) or without COS treatment (red). Samples with 0 or 1 g L^{-1} COS after 72 hour of the primary culture (a and e), with 0 or 3 g L^{-1} COS after 72 hour of the primary culture (b and f), at the end of the 2nd subculture (G2) (c and g), and at the end of the 3rd

subculture (G3) (d and h), were collected. Significant differences in LDA scores ($P < 0.05$) were produced among classes (Kruskal-Wallis test) and between subclasses (Wilcoxon’s test). The threshold logarithmic LDA score was 2.0.

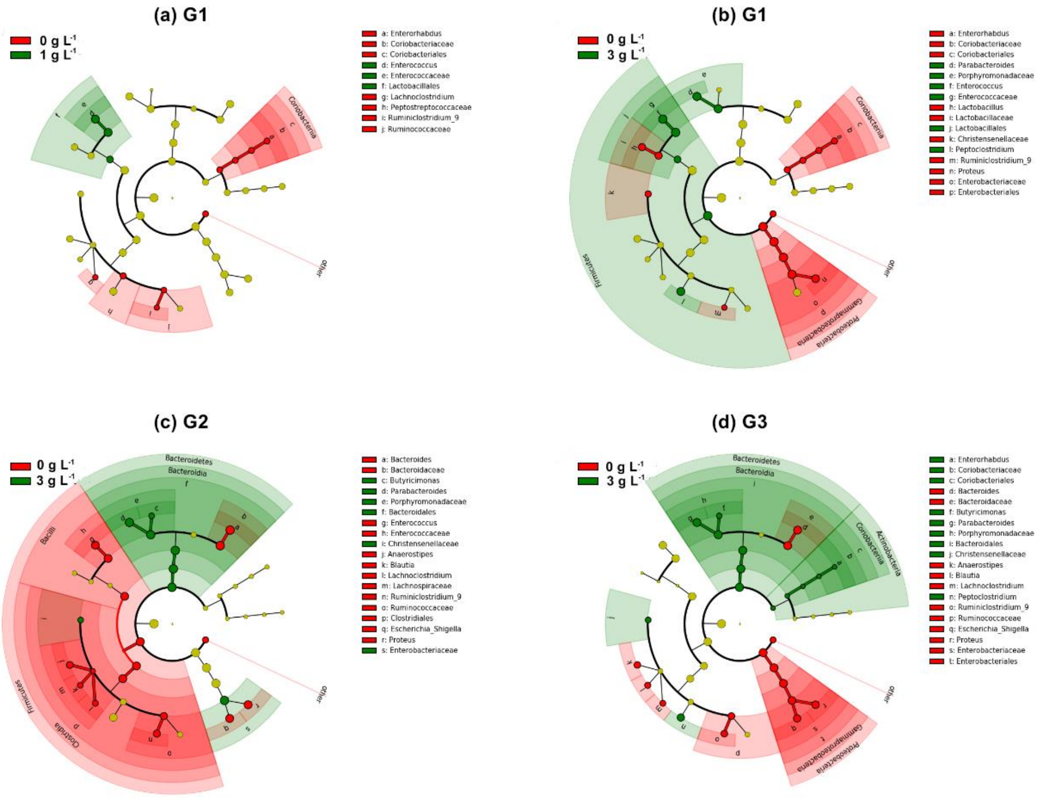

FIGURE S7: A cladogram showing the differences in relative abundance of taxa at four groups: 0 versus 1 g L^{-1} COS, (a), 0 versus 3 g L^{-1} COS, (b) at 72 hour of the primer culture, 0 versus 3 g L^{-1} COS at the end of 2nd subculture (c), 0 versus 3 g L^{-1} COS at the end of 3rd subculture (d). The plot was generated using the online LEfSe project. A taxonomic comparison of the bacterial communities in samples with or without COS treatments in the sample with COS (green) or without COS treatment (red). Yellow circles represent taxon with non-significant differences between two groups.

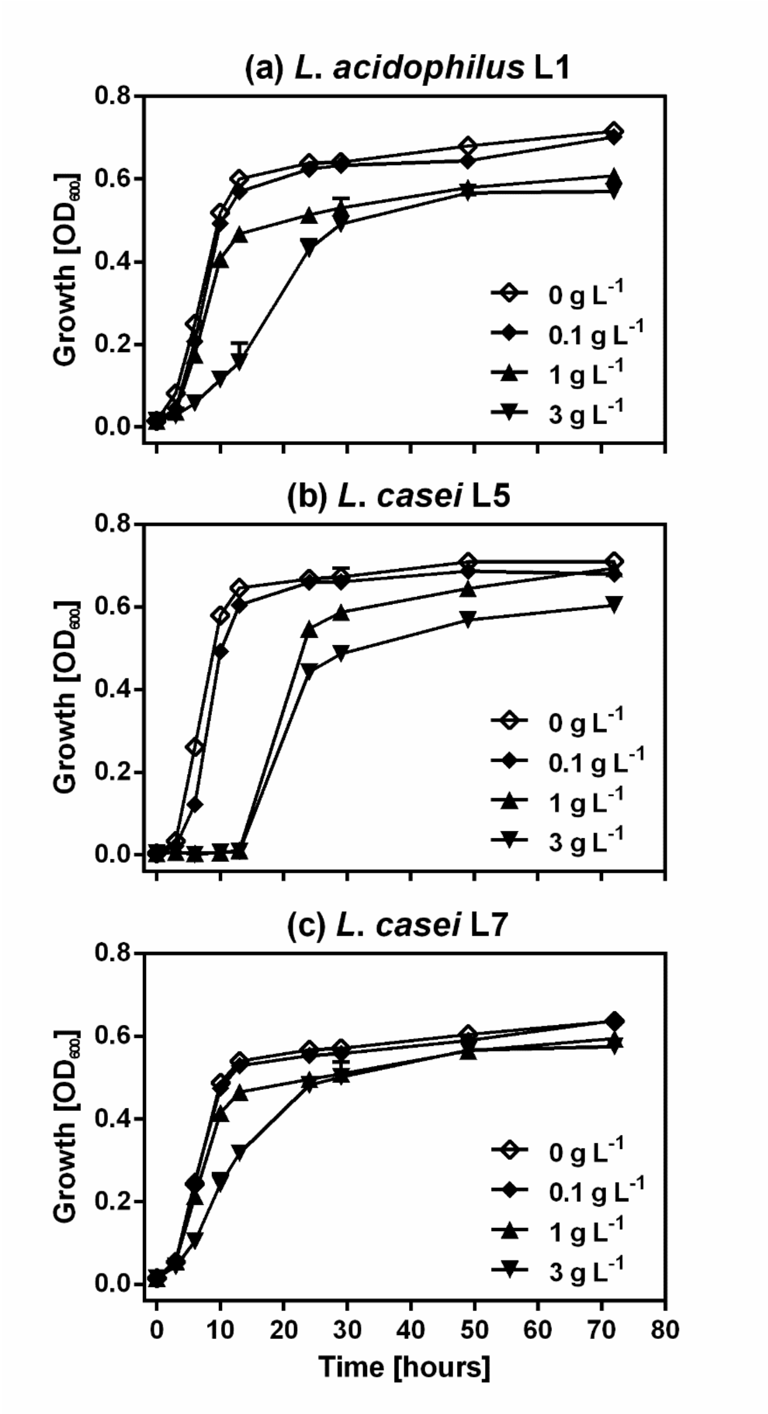

FIGURE S8: Effect of COS on the growth of lactic acid bacteria strains at different cultivation times. Data are represented as the means ± SD (n=3).
